# Neural Encoding of Pitch Direction Is Enhanced in Musically Trained Children and Is Related to Reading Skills

**DOI:** 10.3389/fpsyg.2019.01475

**Published:** 2019-07-24

**Authors:** Vesa Putkinen, Minna Huotilainen, Mari Tervaniemi

**Affiliations:** ^1^ Cognitive Brain Research Unit, Faculty of Medicine, University of Helsinki, Helsinki, Finland; ^2^ TURKU PET Centre, University of Turku, Turku, Finland; ^3^ Faculty of Educational Sciences, University of Helsinki, Helsinki, Finland

**Keywords:** mismatch negativity, musical training, P3a, reading, brain development

## Abstract

Musical training in childhood has been linked to enhanced sound encoding at different stages of the auditory processing. In the current study, we used auditory event-related potentials to investigate cortical sound processing in 9- to 15-year-old children (*N* = 88) with and without musical training. Specifically, we recorded the mismatch negativity (MMN) and P3a responses in an oddball paradigm consisting of standard tone pairs with ascending pitch and deviant tone pairs with descending pitch. A subsample of the children (*N* = 44) also completed a standardized test of reading ability. The musically trained children showed a larger P3a response to the deviant sound pairs. Furthermore, the amplitude of the P3a correlated with a pseudo-word reading test score. These results corroborate previous findings on enhanced sound encoding in musically trained children and are in line with studies suggesting that neural discrimination of spectrotemporal sound patterns is predictive of reading ability.

## Introduction

A vast number of event-related potential (ERP) studies have found evidence for enhanced neural sound processing in musicians. One of the most frequently used EPR components in this framework is the mismatch negativity (MMN) (for a review, see Putkinen and Tervaniemi, 2018). The MMN is elicited by infrequent “deviant” sounds that differ from the preceding frequent “standard” sounds in some way (Kujala et al., 2007; Näätänen et al., 2007). According to influential theoretical accounts, the MMN is a cortical correlate of a prediction error that occurs when an incoming sound (the deviant) disconfirms predictions that the auditory system has automatically created on the basis of the preceding input (the standards) (Näätänen et al., 2007; Winkler et al., 2009). MMN studies have shown that, when compared to non-musicians, musicians show earlier or larger MMN responses to violations of different spectral, temporal, and spatial regularities (Koelsch et al., 1999; Tervaniemi et al., 2001; Fujioka et al., 2004; van Zuijen et al., 2005).

During the last decade, MMN studies have also begun to explore how such differences between musically trained and untrained individuals emerge with accumulation of musical experience in childhood. Cross-sectional MMN studies indicate that musically trained children show enhanced neural discrimination of pitch of violin tones (i.e., their main instrument; Meyer et al., 2011) and major vs. minor chords (Virtala et al., 2012) as well as pitch and voice-onset time changes in speech sounds (Chobert et al., 2011). A longitudinal study in 8- to 10-year-old children who were randomly assigned to music or painting classes (Chobert et al., 2014) found that MMNs to changes in syllable frequency, duration, and voice-onset time increased in amplitude after 12 months of training in the music group but not in the painting group. We have conducted a longitudinal study where we recorded the MMN and other neural markers of sensory processing and higher order cognitive functions in children who play a musical instrument and have attended a public elementary school with heavy emphasis on music in the curriculum, and children who do not play a musical instrument and are matched to the music group with regard to age and socioeconomic status (Putkinen et al., 2014a,b, 2015; Saarikivi et al., 2016; Putkinen and Saarikivi, 2018). In one of these studies (Putkinen et al., 2014b), the amplitude of the MMN elicited by minor chord deviants presented among major chord standards increased more in the music group than in the control group between the ages of 7 and 13 years. In another study (Putkinen et al., 2014a), we found that the MMNs obtained to melody, rhythm, timbre, and tuning deviants increased more in amplitude during the follow-up period. Neither study found evidence of pre-training differences between the groups in neural sound discrimination.

In addition to the enhanced MMN, we found that a P3a-like response elicited by minor chord deviants increased in amplitude with age in the music group but not in the control group (Putkinen et al., 2014a,b) in line with studies in adults that have reported enhanced P3a responses in adult musicians (Nikjeh et al., 2008; Vuust et al., 2009; Seppänen et al., 2012). The P3a is typically interpreted as a marker of involuntary attention capture triggered by salient deviant sounds (Escera et al., 1998; Escera and Corral, 2007). Thus, it appears that certain acoustic changes are more salient for musicians than non-musicians and trigger the attentional orienting more readily in musicians.

Here we report new data from a cross-sectional study conducted partly in the same children and adolescents who participated in our longitudinal studies described above. In the current study, we employed an oddball paradigm consisting of standard tone pairs with ascending pitch and deviant tone pairs with descending pitch (500–750 Hz vs. 750–500 Hz). The aim was to test whether the enhanced processing of changes in musical chords and melodies that we observed previously in the music group would generalize to more basic-level sound processing of pitch order reversal. As a secondary aim, we also tested whether the neural discrimination of the pitch order reversal was associated with reading ability. The rationale was based on the findings that (1) that low-level processing of pitch changes predicts reading abilities (Ahissar et al., 2000; Kujala et al., 2001) and (2) musical training is linked with improved reading skills (Anvari et al., 2002; Moreno et al., 2009) perhaps because musical training improves auditory skills that are necessary for linking speech with its written form. Thereby, we administered standardized test of reading skills in a subset of the children and tested whether the MMN and the P3a elicited would predict their test performance.

## Materials and Methods

### Subjects

Eighty-eight children and adolescents participated in the ERP experiment. The music group (*N* = 41, age range: 8.75–15.92, 25 girls) consisted of children and adolescents who had started playing a musical instrument approximately at age 7 and had attended a public elementary school that integrates musical training (instrument lessons, orchestra practice, music theory studies) in the daily curriculum. The control group (*N* = 47, age range: 8.91–15.83, 18 girls) consisted of children and adolescents without formal musical training who had attended a standard elementary school.

A written informed consent for participation in the study was obtained from the children and their parents or guardians before the experiment. The children were rewarded with two movie tickets for their participation. The experiment protocol was approved by the Ethical Committee of the Department of Psychology (currently the Department of Psychology and Logopedics), University of Helsinki, Finland.

### Stimuli

The stimuli in the ERP experiment consisted of tone pairs (*p* ≈ 85%) with ascending pitch and deviant tones pairs with descending pitch (*p* ≈ 15%). For the standard pairs, the fundamental frequencies of the first and the second tone were 500 and 750 Hz, respectively. For the deviant pairs, the order of the tones was reversed. The duration of each tone was 40 ms including 5-ms rise and fall times. The duration of the silent gap between the tones within a pair was 10 ms. The tone pairs were presented with the stimulus onset asynchrony of 600 ms. All tones were composed of the fundamental and two upper partials and were presented at an intensity of ~60 dB (SPL) through headphones (Sony Dynamic Stereo Headphones, MDR-7506). The stimuli were presented in a pseudorandom order so that two deviant pairs were never presented in succession.

### Procedure and EEG Recording

The duration of the experimental session was approximately 2 h and included five different electroencephalography (EEG) experiments conducted in a counterbalanced order, and a break midway through the session (the four additional experiments are reported elsewhere). EEG was recorded in an electrically shielded and soundproof room while the subjects watched a captioned movie with the sound turned off. They were asked to ignore the sounds and to avoid unnecessary movement.

EEG was recorded using a BioSemi Active-Two system with a sampling rate of 512 Hz from 64 active electrodes mounted on a BioSemi headcap. Additional electrodes were placed below and at the outer canthus of the right eye for monitoring eye movements and blinks, and at the right and left mastoids for offline re-referencing of the data.

### Tests of Reading Skills

Reading ability was measured with a standardized test (Nevala et al., 2006) that involved reading aloud a list of 30 words and a list 30 pseudo-words. The subjects were instructed to read each list as fast and accurately as possible. A composite score incorporating both the completion time and the number of errors was computed separately for the words and pseudo-words for each child. Because of the long duration of the ERP experiment, we only tested the children who were 13 years or older out whom 44 completed both the EEG recording and the reading task (music group: *N* = 20, 12 girls; control group: *N* = 24, 10 girls).

### EEG Data Preprocessing

The data were analyzed using BESA 5.1 software. Data obtained at noisy electrodes were interpolated and the automatic artifact correction system implemented in BESA was applied to remove artifacts related to eye blinks and saccades. The data were filtered with a bandwidth of 1–20 Hz and epoched from −100 to 500 ms relative to stimulus onset. Epochs with voltage changes exceeding ±100 μV were excluded. The epochs were averaged separately for the deviant and standard sounds and re-referenced to the average of the mastoid channels and baseline corrected (−100 to 0 ms).

### Statistical Analyses

Mean response amplitudes were calculated for the deviants and standards over 80-ms time windows centered at the latencies of 200 ms (“MMN”) and 275 ms (“P3a”). Based on visual inspection of the scalp distribution, we chose the channels FC3, FCz, FC4, C3, Cz, and C4 for the analysis of the response amplitudes. The amplitudes were analyzed with a repeated measures ANOVA with Group (music vs. control) as a between-subjects factor and Stimulus (deviant vs. standard) and Left-Middle-Right (FC3 and C3 vs. FCz and Cz vs. F4 and FC4) and Frontal-Central (FC3 and FCz and FC4 vs. C3 and Cz and C4) as within-subjects factors. Greenhouse-Geisser correction was used when the sphericity assumption was violated.

Performance in the reading tasks was analyzed by fitting a linear model where reading score was predicted with age, gender, and group. Partial correlations (controlling for the effects of age and group) were calculated between the reading scores and the response amplitudes at the channels where the deviant-minus-standard amplitude was the largest for each response, i.e., at FC4 for the MMN and at Cz for the P3a.

## Results

**Figure 1:**
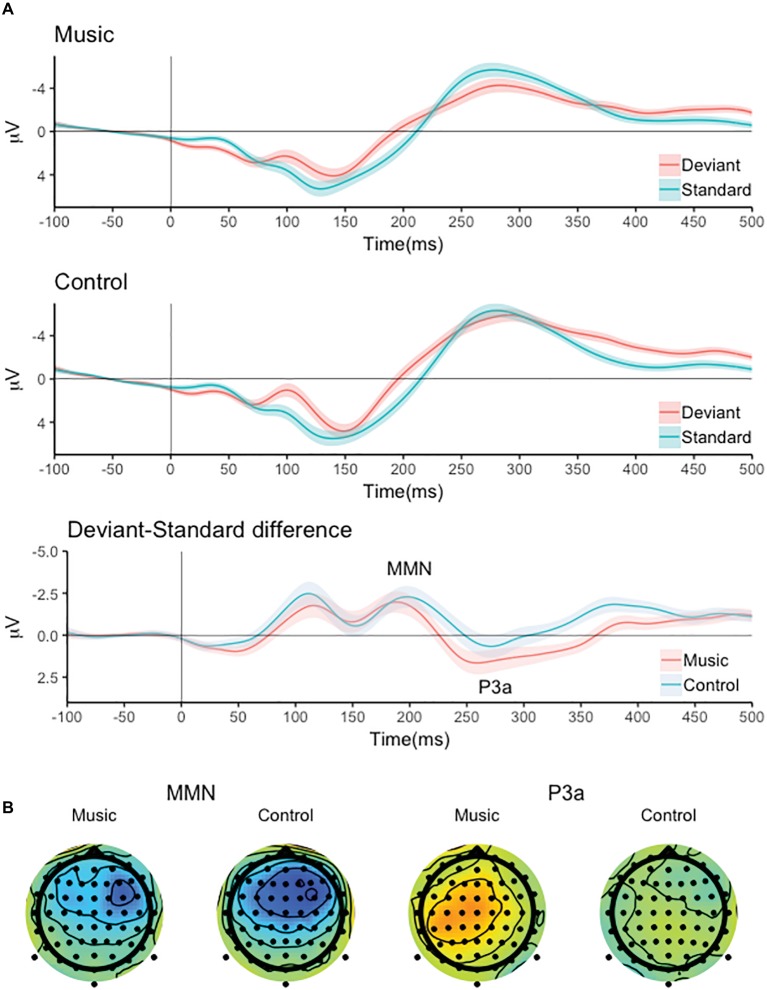
**(A)** The responses to the standard and deviant tone pairs, the deviant-minus-standard difference signals and **(B)** the scalp distribution of the MMN and P3a-like responses for the music and control group.

The responses to the standard and deviant tone pairs and the deviant-minus-standard difference signals are shown in Figure 1. The deviant tone pairs elicited a two-peaked negative response, the latter of which was interpreted as the MMN based on its latency and was chosen for further analysis (Figure 1B). The MMN-like response was followed by a P3a-like positive deflection.

### Event-Related Potential Amplitudes

The responses to the standard and deviant tone pairs, the deviant-minus-standard difference signals at Cz and the scalp distributions of the MMN and P3a are shown in Figure 1.

The deviant pairs elicited a significant MMN response (main effect of stimulus: *F*(1,86) = 42.024, *p* < 0.001) which was largest over the frontal channels and the right hemisphere (Stimulus × Frontal-Central × Left-Midline-Right interaction: *F*(2,172) = 8.419, *p* < 0.001, all pairwise comparisons of deviant-standard difference at channel F4 vs. other channels, *p* < 0.05). There was no significant group difference MMN amplitude (main effect of Group and all interaction involving the Group factor, *p* > 0.05).

The deviant pairs also elicited a significant P3a-like response (main effect of stimulus: *F*(1,86) = 6.500, *p* < 0.05) that was differentially distributed over the left, central and right channel locations (Stimulus × Left-Midline-Right interaction: *F*(2,172) = 4.876, *p* < 0.05). Bonferroni corrected pairwise comparisons of the deviant-minus-standard amplitudes indicated that the P3a was smallest at the right channels (Figure 1).

There was also significant Group × Stimulus × Frontal-Central × Left-Midline-Right interaction (*F*(2,172) = 4.448, *p* < 0.05) indicating that the P3a amplitude differed between the groups at some electrodes included in the analysis. Bonferroni corrected pairwise group comparisons of the deviant-standard amplitudes indicated that the P3a was larger in the music group than in the control group at channels Cz, C3, Cz, and F4 (*p* < 0.05).

### Reading Test Performance

**Figure 2:**
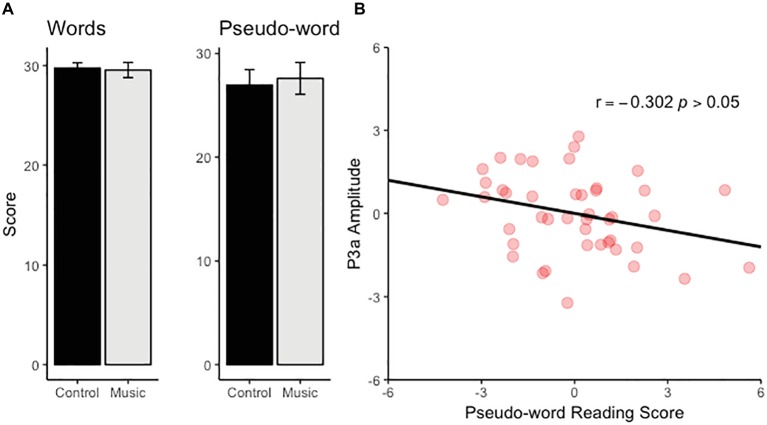
**(A)** The bar charts illustrate the scores for the word and pseudo-word reading task for the music and control group. **(B)** The scatter plot illustrates the relationship between pseudo-word reading score and P3a amplitude (the effects of age and group membership has been controlled and therefore values have the mean 0).

Performance in the pseudo-word subtest improved with age (*b* = 0.504, *t*(41) = 2.241, *p* < 0.05) whereas no effect of age was found for the word subtest (*p* > 0.30). No group difference was found for either subtest (Figure 2) although there was a trend toward a slightly higher scores in the music group than the control group in the pseudo-word subtest (*p* < 0.08). There was no significant correlation between the amplitude of MMN and the performance in either subtest. In contrast, the P3a amplitude at Cz correlated negatively with the score in the pseudo-word subtest (partial *r*(42) = −0.302, *p* < 0.05, controlling for the effects of age and group membership) (Figure 2).

## Discussion

The current study investigated whether musically trained children show facilitated preattentive neural discrimination of pitch order in tone pairs as indexed by the MMN and the P3a. Indeed, we found evidence for enhanced neural discrimination of ascending and descending tone pairs in musically trained children and adolescents. Namely, the music group showed a stronger P3a-like response to reversal of tone order. The MMN, in contrast, did not significantly differ between the groups. Interestingly, the amplitude of the P3a-like response correlated with performance in a pseudo-word reading task in line with the idea that elementary sound processing is linked with reading ability (Ahissar et al., 2000).

The lack of significant group difference in the MMN amplitude is in contrast with a large number of studies that have found enhanced MMN responses in musically trained adults and children (Putkinen and Tervaniemi, 2018). It is noteworthy that the same children who participated in the current study also took part in our previous studies that found stronger MMNs in the musically trained children for musical chords (Putkinen et al., 2014b) and various musically relevant changes in piano melodies (Putkinen et al., 2014a). These results suggest that musical training is associated with enhanced discrimination, as indexed by the MMN, of changes in complex, musically relevant sounds whereas “lower level” sound discrimination investigated in the current study appears not to differentiate musically trained and non-trained children (although strictly speaking the statistical analyses employed in the current study cannot provide evidence for the null hypothesis that the groups do not differ in MMN amplitude). In line with this, our previous study found no evidence for enhanced MMN in the musically trained children for frequency, duration, intensity, and gap deviants of simple tone stimuli (Putkinen et al., 2014b).

The enhanced P3a-like response in the music group nonetheless indicates that the children in the music group were more sensitive to the deviant tone pairs than those in the control group. This result is in line with previous studies in adults and children that have reported enlarged P3a-like responses in musically trained adults and children (Nikjeh et al., 2009; Vuust et al., 2009; Seppänen et al., 2012; Putkinen et al., 2014b). This result indicates that even though the initial change detection reflected by the MMN did not differentiate the groups, a subsequent sound processing stage – possibly related to attention capture – was triggered more strongly in the musically trained children than in the untrained ones.

Interestingly, the MMN was largest and the P3a smallest over the right hemisphere. Although the scalp distribution of ERPs is not related to the location of their neural sources in a straightforward manner, the rightward asymmetry in MMN distribution dovetails with studies showing a stronger involvement of right than left auditory cortical regions in processing of pitch direction (Johnsrude et al., 2000; for reviews see Tervaniemi and Hugdahl, 2003; Zatorre and Gandour, 2008). The leftward asymmetry of the P3a-like response is also intriguing given that this response correlated with reading performance.

The correlation between P3a-like response and reading performance was found for the pseudo-word subtest but not the word reading task. The link between neural sound processing and reading skills might be more robust for pseudo-words than for words because pseudo-word reading requires the conversions of previously unseen letter strings to speech and, arguably, taps into phonetic processing more directly than more automated reading of known words. Notably the participants in the reading tests were the oldest participants of the current study (13 and 15 years) and fully fluent in reading their native language. In fact, due to full orthographic transparency of Finnish alphabet, it is commonly considered as one of the easiest languages for learning to read. Thus, the pseudo-word test may be more sensitive than the word reading test to the processes that govern reading fluency in early adolescence.

Previous studies on examining the relationship between neural sound discrimination indexed by the MMN and the P3a have focused on comparing subjects with reading difficulties and readers in the typical skill range (Kujala et al., 2003; Corbera et al., 2006). It should be noted that these studies have reported smaller responses in adult dyslexic subjects and thereby suggest an opposite correlation between P3a amplitude and reading performance to the one found in the current study. Furthermore, the lack of statistically significant correlation between the MMN and reading skills is seemingly in contrast with previous studies that have reported smaller MMNs in subjects with reading difficulties when compared to those whose reading skills are in the typical range (e.g., Kujala et al., 2001). The reason for this discrepancy is unclear but may originate from differences in the subject samples: It may be more difficult to detect a significant relationship between the MMN and reading ability in typical readers than in a sample that includes a wider range of reading skills. Our P3a results nonetheless offer further support for the notion that elementary neural processing of pitch is associated with reading performance in childhood and adolescence even in typical readers. The negative correlation between the P3a and reading performance could reflect the negative impact of heightened distractibility on reading skills.

Previous studies indicate that musical training is associated with improved readings skills (Butzlaff, 2000). It has been hypothesized that by improving basic auditory processing such as frequency and duration discrimination, musical training may also benefit higher order phonological processing that is critical for reading acquisition (Patel, 2011). The current study, however, did not find clear evidence for improved reading skills in the music group although there was a trend toward a higher score in the music group for the pseudo-word subtest. This suggests that the null result might be due to insufficient statistical power stemming from the relatively small sample size (note that, as mentioned above, only oldest children 13 and 15 years took part in the reading tests for practical reasons).

In conclusion, the current study indicates that pitch direction processing is enhanced in musically trained children and adolescents. However, this enhancement was not evident for the initial change detection reflected by the MMN but only at a later processing stage reflected by a P3a-like response. Furthermore, we found that the P3a-like response was correlated with performance in a reading task in line with the notion that elementary auditory processing is predictive of reading skills.

## Ethics Statement

This study was carried out in accordance with the recommendations of Ethical Committee of the former Department of Psychology, University of Helsinki, Finland, with written informed consent from all subjects. All subjects gave written informed consent in accordance with the Declaration of Helsinki. The protocol was approved by the Ethical Committee of the former Department of Psychology, University of Helsinki, Finland.

## Author Contributions

MH and MT designed the study. VP analyzed the data. VP, MT, and MH wrote the manuscript.

### Conflict of Interest Statement

The authors declare that the research was conducted in the absence of any commercial or financial relationships that could be construed as a potential conflict of interest.
